# Vitamin B and C levels of homeless patients who visit the emergency department with alcohol ingestion

**DOI:** 10.1186/cc13618

**Published:** 2014-03-17

**Authors:** HJ Lee, JH Shin, E Kang, J Jung, DK Kim

**Affiliations:** 1 SMG-SNU Boramae Medical Center, Seoul, South Korea

## Introduction

Vitamins are essential micronutrients and depletions are reported for chronically ill patients. It is well known that the general nutrition status of the homeless is poor, especially for heavy alcoholics. But there were few data about the actual vitamin status of homeless patients. We want to evaluate the vitamin levels of homeless patients.

## Methods

This study was conducted at a single urban teaching hospital emergency department. We performed a retrospective chart review of blood vitamin B1, B12, B6 and C levels of homeless patients. These vitamins are a common supplement in our center and sometimes blood levels are drawn if the patient is drunk, needs i.v. hydration and has cachexic features.

## Results

During study periods, vitamin levels were checked for 156 patients. The number of male patients was 146 (94%) and the mean age was 50 ± 10.2. Vitamin C levels were 15.8 ± 1.3 mg/l. For 84 patients, levels of vitamin C were decreased. For vitamin B1 (152 ± 7.2 nmol/l), vitamin B12 (725.5 ± 35.4 pg/ml), and vitamin B6 (50.3 ± 5.5 ng/ml), there were three, two and 23 patients below the reference ranges respectively. See Table 1.

**Table 1:** 

(%)	Mean (SD)	Reference range	Lower than reference (%)	Higher than reference
Vitamin B1	1 58.1 (80.2;	59 to 213	3.1	13.8
Vitamin B12	741.5 (432.2)	200 to 950	2.7	22.6
Vitamin B6	51.9 (53.8)	20 to 202	23.1	1.9
Vitamin C	15.57 (13.4)	26.1 to 84.6	82.7	0

## Conclusion

The level of vitamin C was markedly decreased. Replacement of vitamin C should be considered for the homeless who visit the emergency department after alcohol ingestion.

